# Simultaneous Removal of Heavy Metals (Cu, Cd, Cr, Ni, Zn and Pb) from Aqueous Solutions Using Thermally Treated Romanian Zeolitic Volcanic Tuff

**DOI:** 10.3390/molecules27123938

**Published:** 2022-06-20

**Authors:** Marin Senila, Emilia Neag, Oana Cadar, Emoke Dalma Kovacs, Ioan Aschilean, Melinda Haydee Kovacs

**Affiliations:** 1 INCDO-INOE 2000, Research Institute for Analytical Instrumentation, 67 Donath Street, 400293 Cluj-Napoca, Romania; emilia.neag@icia.ro (E.N.); oana.cadar@icia.ro (O.C.); dalma.kovacs@icia.ro (E.D.K.); melinda.kovacs@icia.ro (M.H.K.); 2 Zeolites Production S.A., 359 Republicii Street, Rupea, 505500 Brasov, Romania; aschileanioan@gmail.com; 3 Faculty of Civil Engineering, Technical University of Cluj-Napoca, 28 Memorandumului St., 400114 Cluj-Napoca, Romania

**Keywords:** zeolite, heavy metal, aluminosilicate, ICP-OES, adsorbent material, instrumental analysis, kinetic study, ion exchange, chemisorption

## Abstract

Increased concentrations of heavy metals in the environment are of public health concern, their removal from waters receiving considerable interest. The aim of this paper was to study the simultaneous adsorption of heavy metals (Cu, Cd, Cr, Ni, Zn and Pb) from aqueous solutions using the zeolitic volcanic tuffs as adsorbents. The effect of thermal treatment temperature, particle size and initial metal concentrations on the metal ions sorption was investigated. The selectivity of used zeolite for the adsorption of studied heavy metals followed the order: Pb > Cr > Cu > Zn > Cd > Ni. The removal efficiency of the heavy metals was strongly influenced by the particle sizes, the samples with smaller particle size (0–0.05 mm) being more efficient in heavy metals removal than those with larger particle size (1–3 mm). Generally, no relevant changes were observed in heavy metals removal efficiency for the treatment temperatures of 200 °C and 350 °C. Moreover, at a higher temperature (550 °C), a decrease in the removal efficiencies was observed. The Cd, Zn, Cu, Cr, Zn and Ni sorption was best described by Langmuir model according to the high values of correlation coefficient. The pseudo-first-order kinetic model presented the best correlation of the experimental data.

## 1. Introduction

In recent years, there have been increasing public health and environmental concerns related to the contamination with heavy metals [1]. The main sources of these elements in the environment comprise mostly mining, smelters, and other metal-based industrial operations [2]. Metals such as Cd and Pb have no known biological functions and are considered non-essential elements, being toxic even at very low concentrations. Cd affects the respiratory, reproductive, and skeletal organ systems, Pb disturbs the nervous system, while above a certain dose, both Cd and Pb become carcinogenic [3]. Other metals such as Cu, Cr, Ni, Zn have various biochemical and physiological functions, being essential elements. However, elevated concentrations of these elements become toxic [4,5,6]. High Cu concentration in the human body causes damage to internal organs and anemia, while high Cr concentration can affect the liver [7]. Moreover, the metals are non-biodegradable and persistent, and their high toxicity risk is related to the accumulation in soil, water, and living organisms [8]. As a consequence, maximum allowable limits are established for these elements in the environmental factors, and they should be removed from contaminated wastewater before discharging into the environment [9]. Accordingly, numerous methods have been developed to remove heavy metals from water [10]. Of these, the adsorption is known to be the most efficient method, due to its low-cost, high efficiency, reusability, and easy operation [11,12].

The adsorbent materials are characterized by a well-developed porous structure, large specific surface area, and thermal stability. Zeolites are hydrated aluminosilicates of the alkali or alkaline earth metals (Na, K, Mg, Ca) having porous crystalline structures and well-defined channels or cavities [13]. The primary unit is a tetrahedral complex containing Si^4+^ coordinated with 4 O^2−^ ions. The isomorphous substitution of Si^4+^ by Al^3+^ cation in zeolites origins a negative charge in their extended framework [14]. Consequently, the natural zeolites appear as cation exchangers since they have a negative charge on the surface. This negative charge is compensated mainly by the exchanging cations of Na^+^, K^+^, Mg^2+^, and Ca^2+^ that can be substituted by other cations (e.g., cations of heavy metals from contaminated environments) through an ion-exchange mechanism [15,16,17,18]. 

The rock type that contains zeolites is the zeolitic volcanic tuff, while the most abundant mineral from this family is clinoptilolite, characterized by a high cation exchange capacity (CEC) [19]. Due to its relatively high quantity in nature, clinoptilolite was intensively studied as potential ion-exchange material in different industrial, agricultural and environmental applications [20,21,22,23]. Moreover, because they are relatively abundant and widespread in nature, zeolites are cost-effective and accessible materials [24].

The previous studies on the metal adsorption by natural zeolites showed variable results, probably due to the dissimilar CEC, as a consequence of the different chemical composition, structure, pore volumes, or surface-area [25]. Also, the effect of thermal treatment of zeolites on the adsorption rate of metal cations, as well as the effect of competitive adsorption from multielement solutions, is not well known. So, for high removal efficiency, more in-depth experiments on the natural zeolites from different deposits with specific characteristics and their pretreatment are necessary. The aims of this paper are: (1) to characterize the thermally treated zeolitic tuff from the Racoș quarry; (2) to investigate the influence of thermal treatment, contact time, and initial concentrations of metals in solution on the capacity of natural zeolite with two different particle sizes to remove Cu, Cd, Cr, Ni, Zn and Pb from multicomponent contaminated aqueous solutions; (3) to evaluate the contribution of ion exchange and chemisorption processes to the total immobilization of heavy metals in zeolites.

## 2. Results and Discussion

### 2.1. Characteristics of Thermally Treated Volcanic Tuffs

The chemical composition (wt. %) in terms of the major oxides and other components of the two different particle size zeolites (1.0–3.0 mm—NZ1 and 0–0.05 mm—NZ2) thermally treated at 200 °C, 350 °C, and 550 °C is presented in Table 1.

In all cases, the relative standard deviations (RSD%) for the parallel determinations were <5%. As presented in Table 1, the measured Si/Al ratio was >4 and the content of alkaline cations (Na + K) were higher than the content of Ca, indicating the presence of clinoptilolite as a major constituent in the analyzed volcanic tuff. The concentrations of major oxides were generally almost unaffected by crushing and heating treatment, and the differences between the contents of each oxide in different samples, expressed as Coefficients of Variation (CVs), were below 10%. A small decrease in Al_2_O_3_ concentrations is observed in the samples treated at 550 °C, indicating the start of a dealumination process [26].

Dealumination (removing Al atoms) from the zeolitic framework arises at high-temperature treatments, probably caused by the breaking of Si–O(H)–Al bonds [27]. Even though the dealumination mechanism has not been entirely understood, it was reported that the treatment of zeolite at high temperatures leads to the formation of silanol defects. The term “defect” in a zeolite framework refers to the presence of silanol (SiOH) groups [28]. Under severe conditions, even Si atoms can be removed together with the Al atoms from the framework leading to structural defects that reduce the framework stability and thus in amorphization of the structure [28].

**Figure 1:**
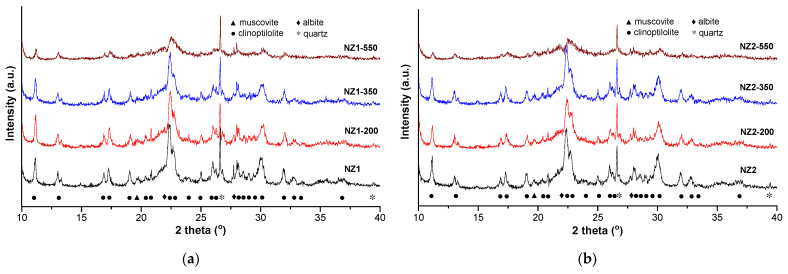
X-ray diffraction patterns of the initial and zeolitic tuff thermally treated at 200 °C, 350 °C, and 550 °C: (**a**) NZ1 and (**b**) NZ2.

According to XRD analysis, the used zeolitic tuff contains Ca-clinoptilolite (PDF 00-047-1870) as the main crystalline phase, accompanied by muscovite (PDF 00-007-0025), quartz (PDF 00-005-0490) and albite (PDF 00-019-1184) (Figure 1). The low amorphous content attributed to the presence of quartz and kaolinized volcanic ash tuff is indicated by the hump in the region 2θ = 18–25° [28,29].

The applied thermal treatment led to a slightly decrease of the clinoptilolite diffraction lines intensities, and thus to a decreasing of crystallinity degrees of the zeolite samples, mainly for the samples with small particle size (0–0.05 mm), as follows: NZ1 (69.1%) > NZ1-200 (67.0%) > NZ1-350 (64.7%) > NZ1-550 (54.4%), and NZ2 (68.5%) > NZ2-200 (67.2%) > NZ2-350 (64.3%) > NZ2-550 (48.4%), respectively. Similar crystallinity loss by thermal treatment was reported for a natural clinoptilolite zeolite from Turkey [29]. The structure damage at 550 °C is indicated by the decrease of clinoptilolite peaks, which are more visible for the main peak at 22.4°. However, the complete amorphization of the crystalline structure does not occur until 550 °C. The thermal stability up to 550 °C of the investigated zeolitic tuff sample can be attributed to the reversible dehydration that arises with slight or no modification of the crystal structure [30]. At the same time, in all cases, the intensity and position of the peak attributed to crystalline quartz (SiO_2_) at 2θ = 26.6° did not change with increasing thermal treatment temperature.

The concentrations of heavy metals (Cu, Cd, Cr, Ni, and Pb) were measured in zeolite solid samples after acid microwave digestion, and those released in water at neutral pH (ratio zeolite: water = 1:10), measured by ICP-OES are presented in Table 2.

The Cr concentrations in the volcanic tuff samples were below the limit of quantification (LOQ). The Cu concentrations were in the range of 1.65–2.03 mg kg^−1^, Cr concentrations were in the range of 7.31–8.41 mg kg^−1^, and the concentrations of Ni ranged between 3.21–4.36 mg kg^−1^, while the concentrations of Pb ranged between 4.96–6.78 mg kg^−1^.

In all cases, the CVs for concentrations of each individual metal in different samples were below 10%, while these variations do not show an increasing or decreasing tendency, indicating that they are caused rather by the measurement uncertainty of the analytical method and not due to particle sizes or temperatures applied during the thermal treatment. The concentrations of all the analyzed heavy metals leached from zeolites into ultrapure water at a neutral pH (pH ≅ 7) were below LOQs (4 µg L^−1^ for Cu, 2 µg L^−1^ for Cd, 7 µg L^−1^ for Cr, 8 µg L^−1^ for Ni and Zn, and 10 µg L^−1^ for Pb). This is an important behavior when accounting for the heavy metals concentrations measured in the solution resulted from batch experiments and shows that the studied zeolites do not release these metals in contact with water, in the used experimental conditions.

CEC values measured by the AMAS method varied in the range of 82.8–112.9 meq 100 g^−1^ and showed a small decrease with the increase in the thermal treatment temperature (Table 3). The exchangeable Ca^2+^, the main component of the total CEC values determined by the AMAS method, is followed by exchangeable K^+^ and lower quantities of Na^+^ and Mg^2+^. However, considering the total amounts of Na^+^, K^+^, Ca^2+^ and Mg^2+^ measured after microwave acid digestion, it can be observed that Na^+^ is the most mobile cation, with 84–99% of the total concentration in exchangeable form. The percent of exchangeable Ca^2+^ is in the range of 47–83% of the total amount, the percent of exchangeable K^+^ is in the range of 40–75% of the total amount, while only a very small part of the Mg^2+^ is exchangeable (2.7–7.8%). As a general remark, the increasing temperature used for the zeolites treatment slowly decreased the exchangeability rate of these cations. The theoretical CEC values calculated based on the microwave-assisted acid extractions (242 meq 100 g^−1^ (NZ1), 231 meq 100 g^−1^ (NZ1-200), 246 meq 100 g^−1^ (NZ1-350), 227 meq 100 g^−1^ (NZ1-550) and, respectively, 220 meq 100 g^−1^ (NZ2), 221 meq 100 g^−1^ (NZ2-200), 230 meq 100 g^−1^ (NZ2-350), 248 meq 100 g^−1^ (NZ2-550)) highly exceeded the effective CEC value determined by AMAS method. These results indicate that 35–51% of exchangeable sites are active and can be implied in the exchange processes.

According to the International Union of Pure and Applied Chemistry (IUPAC), the adsorbents are classified based on their sizes of pores into three categories: macropores, with a dimension greater than 50 nm, mesopores with dimensions ranging between 2 and 50 nm and micropores with dimension less than 2 nm [25].

As presented in Table 3, all the analyzed samples contain only mesopores. The surfaces of these pores are linked with active functional groups that contribute to the adsorption process and offer spaces for the sequestration of heavy metals. The surface areas ranged between 33–38 m^2^ g^−1^ showing a slight diminishing in samples treated at higher temperatures. The specific surface area of porous materials is divided into external and internal specific surface areas [31]. By grinding, the external surface area increases, but the internal surface area, which has the most important contribution to the specific surface area in porous materials, is unchanged or even decreases. Thus, if the external surface area increases by grinding, it does not necessarily lead to an increase in the total specific surface area (determined by BET). Similarly, Burris and Juenger [32] reported that the specific surface area does not significantly increase by zeolite milling.

### 2.2. Removal Efficiency (E%) for Heavy Metals Ions from Contaminated Solutions

#### 2.2.1. Zeolite Selectivity for Heavy Metals Sorption and Effect of Contact Time

The selectivity of used natural zeolite in our experiments for the sorption of studied heavy metals decreased as follows: Pb > Cr > Cu > Zn > Cd > Ni. Zamzow et al. [33] reported for the sodium form of clinoptilolite the following order of selectivity: Pb > Cd > Cs > Cu > Co > Cr > Zn > Ni > Hg, which, except Cd, is similar to our results. Belova [14] reported a sorption capacity for natural zeolite from the Yagodnisky deposit in the order Cu > Fe > Ni > Co. The natural zeolites studied by Sprynskyy et al. [34] adsorbed heavy metal ions in the following order: Pb > Cu > Cd > Ni, which is similar to our results. In addition, Hong et al. [35] reported an adsorption capacity trend: Pb > Cu > Ni. According to these findings, it can be concluded that the affinity of clinoptilolite natural zeolite toward different heavy metals is mostly similar, but for some cations, this is related to the zeolites’ specific properties [36].

Generally, the metals’ sorption reached equilibrium even after 5 min of contact time, mainly for the small particle size. Thus, a contact time of 60 min was considered long enough to compare the adsorption behavior of the two types of particle sizes (NZ1 and NZ2).

#### 2.2.2. Influence of Zeolite Particle Size on Removal Efficiency (*E*%)

**Figure 2:**
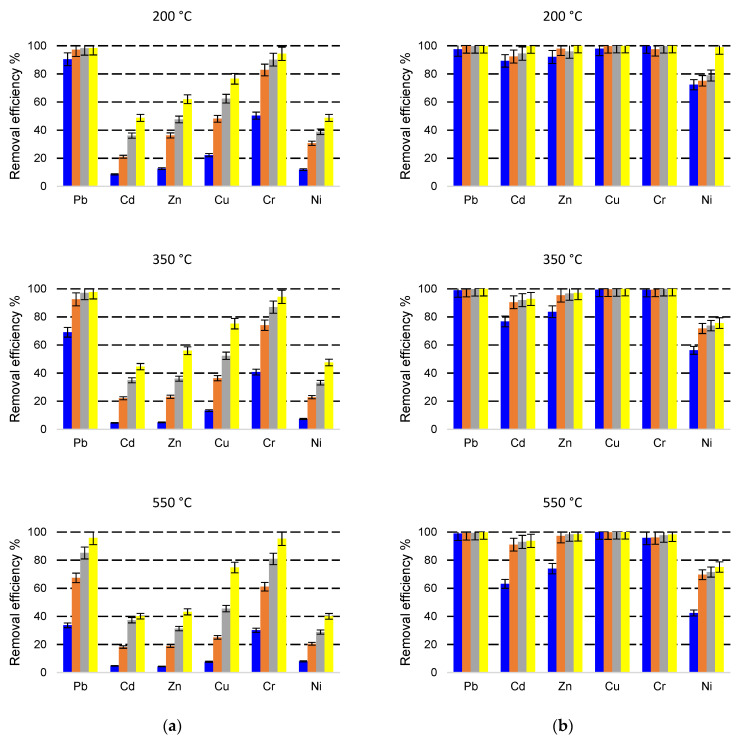
Simultaneous removal of Pb, Cd, Zn, Cu, Cr, Ni with NZ1 (**a**) and NZ2 (**b**) thermally treated at 200 °C, 350 °C, and 550 °C from a multicomponent solution of 5 mg L^−1^ at four different contact times (5 min—blue, 10 min—orange, 30 min—grey, 60 min—yellow).

**Figure 3:**
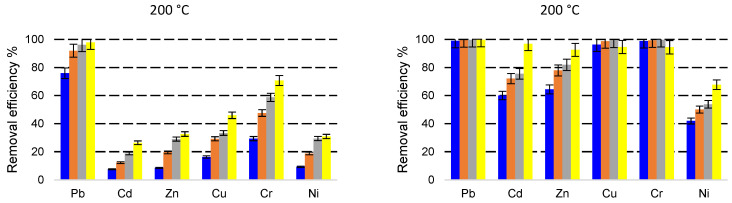

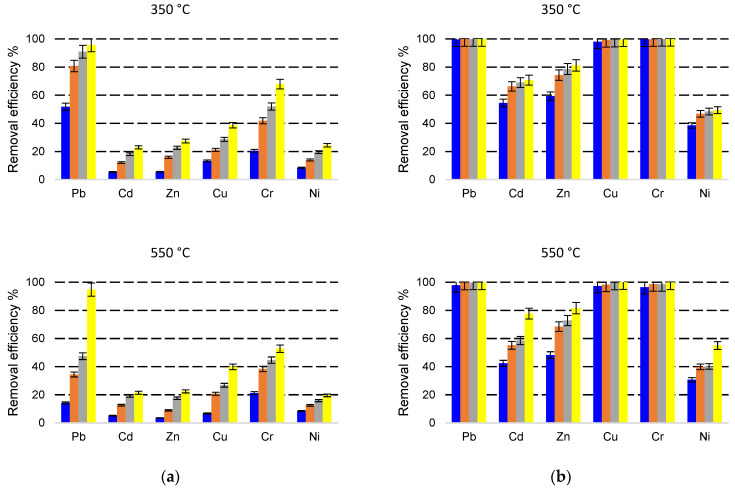
Simultaneous removal of Pb, Cd, Zn, Cu, Cr, Ni with NZ1 (**a**) and NZ2 (**b**) thermally treated at 200 °C, 350 °C, and 550 °C from a multicomponent solution of 10 mg L^−1^ at four different contact times (5 min—blue, 10 min—orange, 30 min—grey, 60 min—yellow).

**Figure 4:**
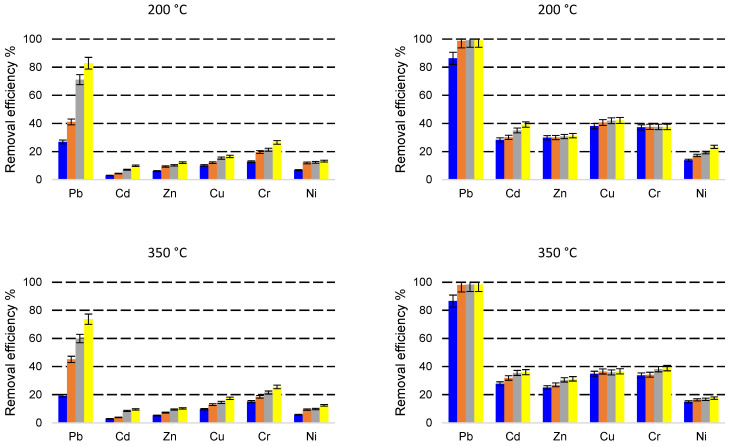

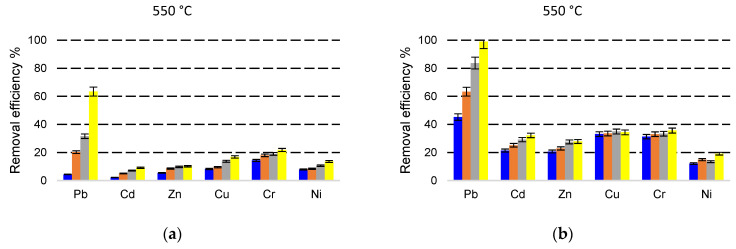
Simultaneous removal of Pb, Cd, Zn, Cu, Cr, Ni with NZ1 (**a**) and NZ2 (**b**) thermally treated at 200 °C, 350 °C, and 550 °C from a multicomponent solution of 30 mg L^−1^ at four different contact times (5 min—blue, 10 min—orange, 30 min—grey, 60 min—yellow).

As shown in Figure 2, Figure 3 and Figure 4, the heavy metals removal efficiencies are strongly influenced by the size of the adsorbent particle. Thus, when the NZ2 samples (particle size 0–0.05 mm) were contacted with the initial solution containing 5 mg L^−1^ heavy metals, the removal efficiencies (*E*%) reached nearby 90–99% after 5 min contact time, except for Ni^2+^ for which *E*% was in the range of 42.4–72.4%. In the case of NZ1 with a particle size of 1–3 mm contacted with the same initial concentration (5 mg L^−1^), the removal efficiencies (*E*%) were generally much lower, only Pb being highly removed from solutions.

According to the results, the removal strongly depends on the particle size of the zeolite, with fine particles much more efficient than coarse particles, because of a greater external surface area available for the sorption of heavy metals, which enhances the adsorption process. Even if the total specific surface area measured by BET does not increase notably for fine particles, the decrease in particle size leads to the increase of external surface area available for the interaction with metal ions in solution and results in shorter diffusion path lengths for sorption. The diffusion of metal ions from the surface to interparticle sites in zeolites is slow due to the interaction of metal ions with the surface functional group (electrostatic attraction, bond formation, etc.) [37].

Furthermore, the diffusion path length of the cations through the adsorbent cations is essentially shortened, which also simplifies the sorption [38]. Pernyeszi et al. [39] also reported that smaller adsorbent particle sizes have better sorption than larger particles due to a synergy between increased specific surface area and active sites.

The removal efficiency (*E*%) was remarkably increased when a smaller particle size (0–0.05 mm) zeolite was used. For the solution with an initial concentration of 5 mg L^−1^, *E*% increased by 1.3, 5.7, 5.6, 3.6, 1.5, and 3.4 times for Pb, Cd, Zn, Cu, Cr and Ni. For the solution with an initial concentration of 10 mg L^−1^, *E*% was improved on average by 1.9, 5.2, 5.7, 4.8, 2.5, and 3.0 times. When the initial concentration was 30 mg L^−1^, *E*% was improved on average by 2.9, 6.1, 3.4, 2.9, 1.9, and 1.8 times.

The increase of *E*% due to the use of fine particle size is clearer at a shorter contact time (5 min). This implies that the fine particle sizes increase the sorption speed, and the system reaches an equilibrium state after a much shorter contact time compared with the larger particles.

#### 2.2.3. Influence of Initial Heavy Metals Concentration in Solution

Concentration is one of the important factors that influence the removal of metal ions. As shown in Figure 2, the heavy metals removal efficiencies are influenced by the initial concentrations of heavy metals, as an effect of saturation of active sites from zeolite surfaces. However, even if the removal efficiencies generally decrease with the increasing initial concentrations, in terms of amounts of adsorbed metals, it can be observed that the increased metal concentration leads to a higher sorption capacity. This can be explained by the higher gradient of concentration between the solution and the adsorbent phase and by the higher number of cations around the active sites of the adsorbent, which increase the probability of adsorption [40]. Taamneh and Sharadqah [40] also reported that the percentage of adsorption of Cd and Cu on natural zeolite decreases by increasing the concentrations of the initial ions.

#### 2.2.4. Influence of Thermal Treatment Temperature on Removal Efficiency (*E*%)

The influence of thermal treatment temperature on zeolite removal efficiency can be observed in Figure 2, Figure 3 and Figure 4. In general, no relevant changes were observed in the removal efficiency for the treatment temperatures of 200 °C and 350 °C. At a higher temperature of 550 °C, small decreases in the removal efficiencies were observed mainly at shorter contact time. These results are correlated with the decreasing of clinoptilolite content observed in the XRD analysis of samples treated at 550 °C.

### 2.3. Amounts of Heavy Metals Ions Sorption from Contaminated Solutions

**Figure 5:**
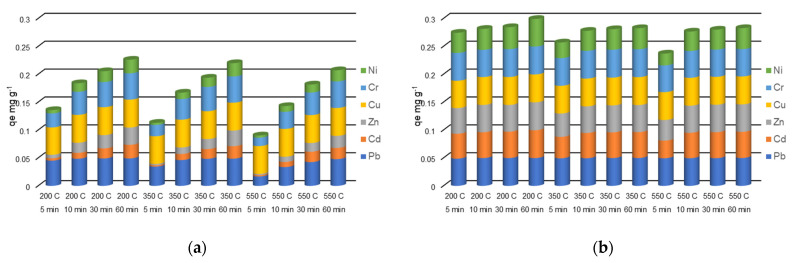
Heavy metals amount in the adsorbent phase *q_e_* (mg g^−1^) with a grain size of 1–3 mm (**a**), and 0–0.05 mm (**b**), thermally treated adsorbed from multicomponent solutions of 5 mg L^−1^ at four different contact times (5, 10, 30, 60 min).

**Figure 6:**
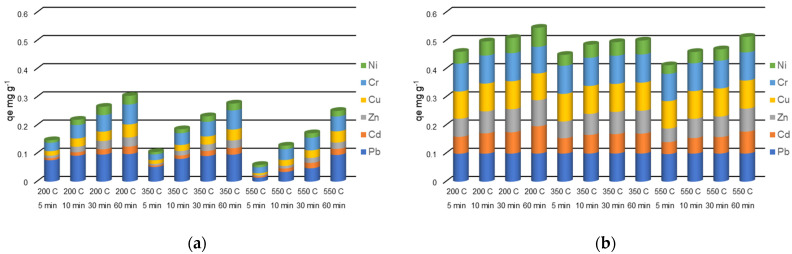
Heavy metals amount in the adsorbent phase *q_e_* (mg g^−1^) with a grain size of 1–3 mm (**a**), and 0–0.05 mm (**b**), thermally treated adsorbed from multicomponent solutions of 10 mg L^−1^ at four different contact times (5, 10, 30, 60 min).

**Figure 7:**
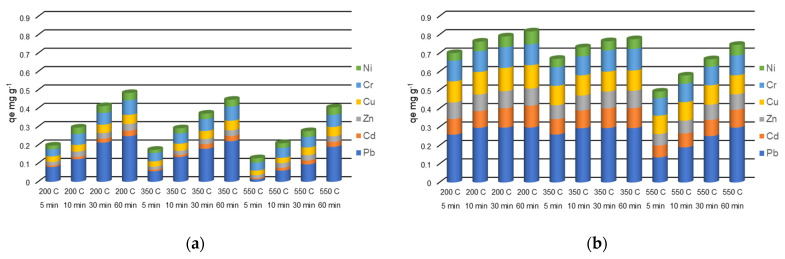
Amount of heavy metals in the adsorbent phase *q_e_* (mg g^−1^) with a grain size of 1- mm (**a**), and 0–0.05 mm (**b**), thermally treated adsorbed from multicomponent solutions of 30 mg L^−1^ at four different contact times (5, 10, 30, 60 min).

The evolution of the metal amounts in the adsorbent phase during the 60 min of experiments from the solutions with different initial concentrations are presented in Figure 5, Figure 6 and Figure 7.

The amounts of heavy metals sorbed into the adsorbent phase, *q_e_* (mg g^−1^) calculated using Equation (1) are strongly influenced by the concentrations of heavy metals in the initial solutions (Figure 5, Figure 6 and Figure 7). In addition, the zeolites particle sizes and metals species play an important role in *q_e_* values. When a solution containing 5 mg L^−1^ of each heavy metal made contact with the particle size 0–0.05 mm zeolites, the *q_e_* values ranged between 0.0488–0.0500 mg g^−1^ Pb, 0.0316–0.0498 mg g^−1^ Cd, 0.0370–0.0500 mg g^−1^ Zn, 0.0490–0.0500 mg g^−1^ Cu, 0.0479–0.0500 mg g^−1^ Cr, and 0.0212–0.0495 mg g^−1^ Ni. Almost the entire amounts of all the heavy metals from solution were sorbed onto the zeolites after the 60 min contact time. The solution containing 5 mg L^−1^ heavy metals mixed with the zeolite with a 1–3 mm particle size led to the *q_e_* values on a more dispersed domain: 0.0168–0.0492 mg g^−1^ Pb, 0.0023–0.0244 mg g^−1^ Cd, 0.0022–0.0310 mg g^−1^ Zn, 0.0038–0.0383 mg g^−1^ Cu, 0.0151–0.0476 mg g^−1^ Cr, and 0.0037–0.0244 mg g^−1^ Ni. In this case, the *q_e_* values were significantly influenced by the contact time.

In the case of solution with an initial concentration of 10 mg L^−1^ heavy metals in contact with a 0–0.05 mm particle-size zeolites, the *q_e_* values ranged between 0.0978–0.0998 mg g^−1^ Pb, 0.0424–0.0969 mg g^−1^ Cd, 0.0482–0.0926 mg g^−1^ for Zn, 0.0946–0.0999 mg g^−1^ Cu, 0.0944–0.0999 mg g^−1^ for Cr, and 0.0307–0.0678 mg g^−1^ for Ni. Almost the entire amounts of heavy metals from the solution are adsorbed onto the zeolites after the 60 min contact time. The values of *q_e_* are, in general, twice that of those in the experiment with the 5 mg L^−1^ initial concentration. When the 10 mg L^−1^ solution was mixed with the 1–3 mm particle-size zeolites resulted in *q_e_* values in the ranges of 0.0142–0.0978 mg g^−1^ Pb, 0.0052–0.0264 mg g^−1^ Cd, 0.0035–0.0326 mg g^−1^ Zn, 0.0068–0.0460 mg g^−1^ Cu, 0.0205–0.0707 mg g^−1^ Cr, and 0.0084–0.0309 mg g^−1^ Ni, with increasing *q_e_* values in time.

The zeolites of 0–0.05 mm particle size contacted with the solution containing 30 mg L^−1^ metals adsorbed between 0.1357–0.2974 mg g^−1^ Pb, 0.0643–0.1179 mg g^−1^ Cd, 0.0622–0.0939 mg g^−1^ Zn, 0.0994–0.1264 mg g^−1^ Cu, 0.0940–0.1168 mg g^−1^ Cr, and 0.0364–0.0700 mg g^−1^ Ni. When the 30 mg L^−1^ solution was contacted with a 1–3 mm particle-size zeolite *q_e_* values were in the ranges of 0.0132–0.248 mg g^−1^ for Pb, 0.0068–0.0298 mg g^−1^ for Cd, 0.0158–0.0365 mg g^−1^ for Zn, 0.0248–0.0525 mg g^−1^ for Cu, 0.0385–0.0795 mg g^−1^ for Cr, and 0.0174–0.0407 mg g^−1^ for Ni.

### 2.4. Isotherm and Kinetic Modeling

**Figure 8:**
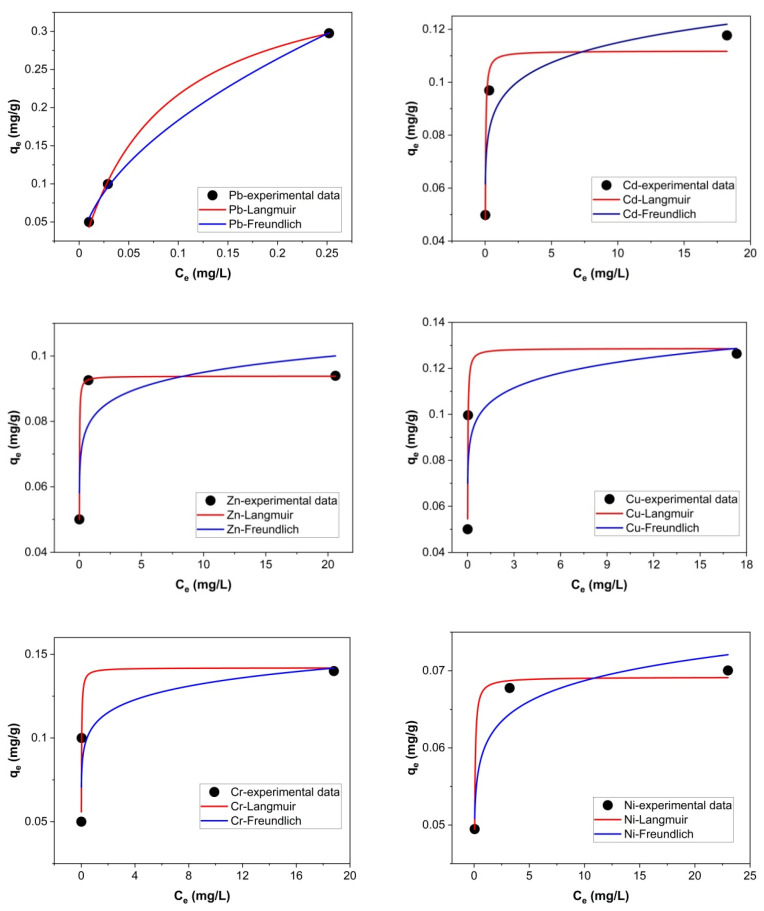
Nonlinear fitting of Langmuir and Freundlich isotherm models for the Pb, Cd, Zn, Cu, Cr and Ni ions sorption onto zeolite with a 0–0.05 mm particle size thermally treated at 200 °C.

The sorption data of Pb, Cd, Zn, Cu, Cr and Ni ions onto zeolite with a 0–0.05 mm particle size thermally treated at 200 °C were analyzed using the nonlinear forms of Langmuir and Freundlich isotherm models. The nonlinear plots of Langmuir and Freundlich isotherm models for Pb, Cd, Zn, Cu, Cr and Ni ions sorption onto zeolite with a 0–0.05 mm particle size thermally treated at 200 °C are presented in Figure 8.

The Langmuir and Freundlich isotherm parameters are given in Table 4.

The best fit was achieved with the Langmuir equation according to the high values of correlation coefficients (R^2^) obtained for Cd (R^2^ = 0.9615), Zn (R^2^ = 0.9999), Cu (R^2^ = 0.9814), Cr (R^2^ = 0.9812) and Ni (R^2^ = 0.9930) sorption. In the case of Pb sorption, the correlation coefficient of Langmuir isotherm is high (R^2^ = 0.9982) and close to that of Freundlich isotherm (R^2^ = 0.9989). The maximum adsorption capacities (*q_max_*_)_ from Langmuir isotherm were 0.393 (Pb), 0.112 (Cd), 0.094 (Zn), 0.129 (Cu), 0.142 (Cr) and 0.069 mg g^−1^ (Ni), respectively. The following series was depicted based R^2^ values obtained from the Langmuir model: Zn, Pb, Ni, Cu, Cr and Cd. The *n* values (Freundlich isotherm model) indicated normal sorption of the metals onto zeolite. According to the obtained R^2^ values, the Freundlich isotherm model cannot adequately describe the sorption of Cd, Zn, Cu, Cr and Ni onto zeolite with a 0–0.05 mm particle size.

**Figure 9:**
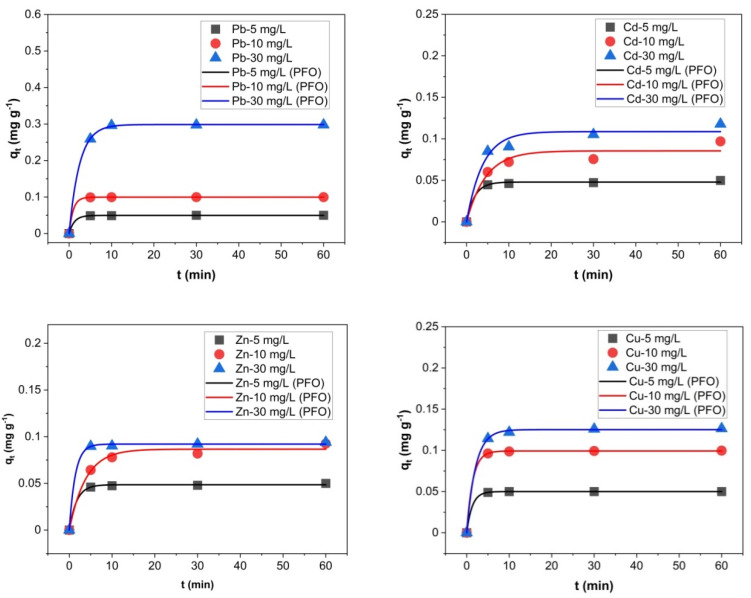

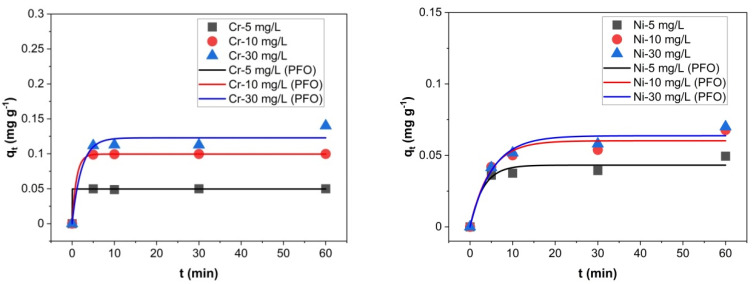
Nonlinear fitting of PFO for Pb, Cd, Zn, Cu, Cr and Ni ions sorption onto zeolite with a 0–0.05 mm particle size thermally treated at 200 °C.

**Figure 10:**
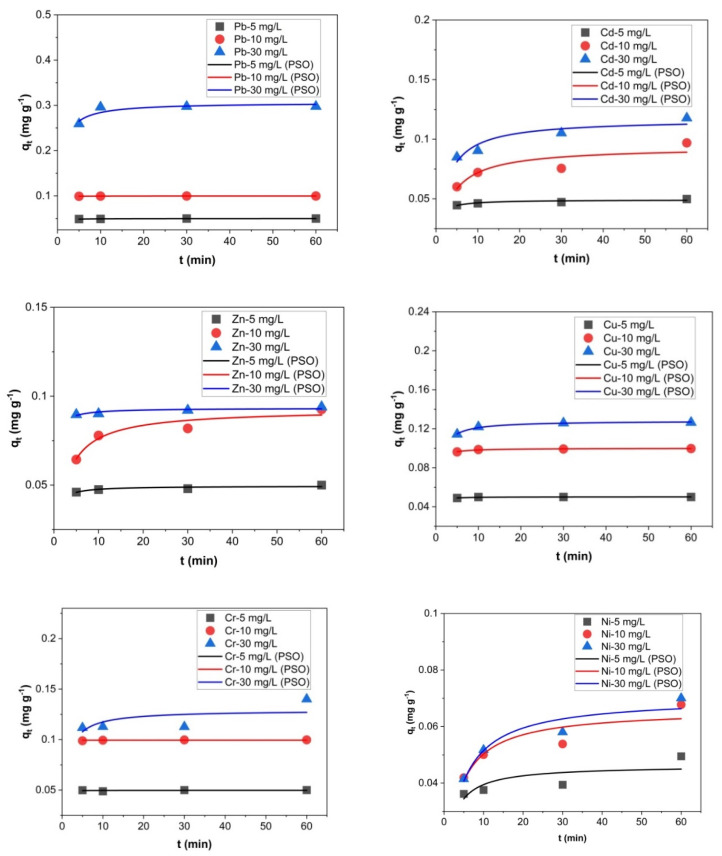
Nonlinear fitting of PSO for Pb, Cd, Zn, Cu, Cr and Ni ions sorption onto zeolite with a 0–0.05 mm particle size thermally treated at 200 °C.

In addition, the experimental results were analyzed using the nonlinear forms of pseudo-first-order (PFO) and pseudo-second-order (PSO) kinetic models. The nonlinear plots of PFO and PSO for Pb, Cd, Zn, Cu, Cr and Ni ions sorption onto zeolite with a 0–0.05 mm particle size thermally treated at 200 °C are presented in Figure 9 and Figure 10, respectively. The PFO and PSO parameters are given in Table 5 for all the studied metals. The experimental *q_e_* values (*q_e_,_exp_*) were very close to the calculated *q_e_*_,_ values (*q_e,calc_*) for PFO and PSO. The R^2^ values obtained from the PFO model were very high compared with the R^2^ values obtained from the PSO model. Thus, the PFO model described the sorption data of Pb, Cd, Zn, Cu, Cr and Ni ions sorption onto the zeolite with a 0–0.05 mm particle size thermally treated at 200 °C better than PSO.

Heavy metal ions can be immobilized by zeolites by two main mechanisms: ion-exchange and chemisorption [41]. Ion exchange involves the substitution of the exchangeable cations (Na^+^, K^+^, Ca^2+^ and Mg^2+^) located in the zeolite crystalline lattice by heavy metals cations from the solution. Since metals retained in zeolite by ion exchange are weakly physically bounded, they can be replaced to form zeolite with NH_4_^+^. Chemisorption is based on the formation of stable inner-sphere complexes by chemical bonds of functional groups (mainly OH^−^) with metal ions outside the hydration envelope [41,42]. In zeolites, ion-exchange processes generally dominate chemisorption, but this depends on the metal species [42]. To evaluate the sorption of metals on zeolites due to the ion-exchange process, the zeolite used for metal ions removal in sorption experiments was mixed with ammonium acetate solution 1 M (ratio 1:50), and the desorbed metals in the extraction solutions were measured using ICP-OES. It was assumed that NH_4_^+^ assures the removal of all metal ions retained by ion exchange, while the heavy metals that remained in the zeolites were retained by chemisorption [42]. According to the results presented in Table 6, the total immobilization of heavy metals in zeolite is caused both by ion exchange and chemisorption, but their contribution depends on the type of metal.

In the case of Cu, ion exchange is by far the most important mechanism of retention (over 85%). In the case of Pb and Cd, about 60–70% of retention is caused by ion exchange; in the case of Zn, the ion exchange and chemisorption have almost equal contributions to its retention, while in the case of Cr and Ni, the chemisorption retention mechanism prevails, but the ion exchange still contributes approximately 41–47% for Cr, and 33–43% for Ni. Krol et al. [41] reported that chemisorption predominates for Cr, chemisorption and ion exchange are almost of equal importance for Cd and Pb, whereas in the case of Ni ion-exchange processes prevail.

## 3. Materials and Methods

### 3.1. Materials

All chemicals used in this study were of analytical reagent grade. Emsure^®^ ACS premium-grade acids HNO_3_ 65%, HCl 37%, and HF 40%, purchased from Merck (Darmstadt, Germany), were used for the sample digestion. Ultrapure water (18 MΩ cm^−1^) obtained from a Millipore Direct Q3 (Millipore, Bedford, MA, USA) was used for dilutions. Standard solutions for external calibration of ICP-OES were prepared by the stepwise dilution of a Merck Millipore CertiPur ICP multi-elemental standard solution IV (23 elements) 1000 mg L^−1^, purchased from Merck (Darmstadt, Germany). The accuracy of the analysis for total metal concentrations in zeolite samples was assessed using CRM BCS-CRM 375/1 soda feldspar (Bureau of Analyzed Samples, Middlesbrough, UK). The recovery percentages of the analyzed elements in CRM were in the range of 86–102%.

### 3.2. Zeolite Preparation and Characterization

Natural zeolite (NZ) material was extracted from a quarry located in Racoș, Brasov County, Romania. The fractions with particle sizes of 1.0–3.0 mm (NZ1) and 0–0.05 mm (NZ2) were produced in the Zeolites Production Rupea Factory by heat treatment at 150 °C, crushing and granulometric separation during the technological process. Both NZ1 and NZ2 fractions were further thermally treated at 200 °C, 350 °C, and 550 °C for 2 h, to obtain the samples NZ1-200, NZ1-350, NZ1-550, and NZ2-200, NZ2-350, NZ2-550, respectively. For the determination of physicochemical and structural characteristics such as major oxides, metals, exchangeable cations content, the sum of exchangeable cations (CEC), XRD, aliquots from NZ1, NZ1-200, NZ1-350, NZ1-550 were ground in the laboratory, using a micronization system (PilotMill-2 FPS1015, Como, Italy) to obtain particle size <0.05 mm. Total surface area, pore radius and total pore volumes were measured on fractions with particle sizes of 1.0–3.0 mm and 0–0.05 mm.

To determine the major elements (Al, Ca, Mg, K, Na, Fe, Mn) and trace metals (Cu, Cd, Cr, Ni, and Pb) in zeolite samples, microwave-assisted acid digestion with an Xpert system (Berghof, Eningen, Germany) was carried out. An amount of 1 g of zeolite sample was digested with a 14 mL mixture of HNO_3_ 65%:HCl 37%:HF 40% (3:9:2, *v*:*v*:*v*) in a three-steps heating program until 200 °C, with a total digestion time of 40 min. After cooling down at room temperature, 20 mL of saturated H_3_BO_3_ were added, then heated again at 160 °C for 15 min, then filtered and diluted with ultrapure water to a final volume of 100 mL. Three parallel determinations were carried out for each sample analysis.

In order to evaluate the possible release of Cu, Cd, Cr, Ni, Zn, and Pb into the water at a neutral pH, 5 g of each zeolite sample was mixed with 45 mL ultrapure water and stirred at room temperature (23 ± 2 °C) for 60 min, filtered through a cellulose filter, then analyzed for metals. The resulting solutions (from zeolite digestion, released into ultrapure water and from batch adsorption experiments) were analyzed for metals using a dual-viewing inductively coupled plasma optical emission spectrometer Optima 5300DV (Perkin Elmer, Norwalk, CT, USA). The seven-point linear calibration curves over the range 0–10 mg L^−1^ element were constructed for each analyzed element. The concentrations of major elements (Al, Ca, Mg, K, Na, Fe, Mn) in the zeolites were converted to oxides using atomic and molecular masses. The SiO_2_ content was determined gravimetrically [26].

The cation exchange capacity (CEC) was determined by measuring using ICP-OES the major cations (K, Na, Ca, and Mg) extracted in ammonium acetate solution 1 M (AMAS method). Total surface area, pore radius and total pore volumes were obtained from N_2_ adsorption–desorption isotherms using the Brunauer–Emmett–Teller (BET) method for total surface area evaluation and Dollimore—Heal model for porosity data. The isotherms were obtained using a Sorptomatic 1990 apparatus (Thermo Electron Corporation, Waltham, MA, USA). The X-ray diffraction (XRD) patterns were recorded at room temperature using a D8 Advance (Bruker, Karlsruhe, Germany) diffractometer with CuKα radiation (λ = 1.54060 Å), operating at 40 kV and 40 mA.

### 3.3. Batch Sorption Experiments

A multicomponent stock solution containing Cu, Cd, Cr, Ni, Zn and Pb 1 g L^−1^ was used to prepare the chosen concentrations by dilution with ultrapure water. The experiments were performed in batch mode, contacting 5 g of zeolites (NZ1-200, NZ1-350, NZ1-550, and respectively, NZ2-200, NZ2-350, NZ2-550) with 45 mL solutions at different Cu^2+^, Cd^2+^, Cr^3+^, Ni^2+^, and Pb^2+^ initial concentrations (5 mg L^−1^, 10 mg L^−1^; 30 mg L^−1^) at a stirring rate of 100 rpm. All the experiments were performed at room temperature (23 ± 2 °C). Samples were taken at established time intervals (5, 10, 30, 60 min) and were filtered before ICP-OES analysis. All the experiments were carried out in triplicate, and the average values were used to calculate the results. The standard deviations were determined at less than 5%.

The heavy metal amounts in the adsorbent phase, *q_e_* (mg g^−1^) were calculated using Equation (1), while the removal efficiency, *E* (%), was calculated using Equation (2) [43]:

(1) $$q_{e}=\frac{\left(C_{o}-C_{e}\right)}{m}\cdot \frac{V}{1000}$$

(2) $$E(\%)=\frac{{(C}_{o}-C_{e})}{C_{o}}\cdot 100$$

where *q_e_* is the heavy metal amounts adsorbed per gram of adsorbent at equilibrium (mg g^−1^), *V* is the volume of solution (mL), *m* is the weight of zeolite (g), *C_e_* is the equilibrium metals concentrations (mg L^−1^) and *C_o_* is the initial metal concentrations (mg L^−1^).

The heavy metal amounts desorbed from zeolite resulted from sorption experiments were measured after desorbing in ammonium acetate solution 1 M and the zeolite: solution ratio of 1:50 [42]. The resulted slurries were mixed at a stirring rate of 100 rpm for 2 h at room temperature, then were centrifuged and filtered. The metal concentrations in the resulted solutions were determined using ICP-OES. Finally, the quantities of metals desorbed from the solid phase were calculated considering the initial amount of zeolite (2 g) and the final volume of the extraction solutions (100 mL).

### 3.4. Adsorption Isotherms and Kinetics

The Langmuir and Freundlich isotherm models were applied to describe the sorption process of Pb, Cd, Zn, Cu, Cr and Ni ions onto the zeolite with a 0–0.5 mm particle size thermally treated at 200 °C and the pseudo-first-order and pseudo-second-order kinetic models were applied to study the adsorption kinetics. The nonlinear forms of the considered models are given in Table 7 [44]. The Langmuir isotherm suggests monolayer sorption onto an adsorbent surface [44]. The Freundlich isotherm suggests that the adsorption occurs onto a heterogeneous surface [44]. The *n* value indicates a normal adsorption when *n* < 1, cooperative adsorption when *n* > 1 and a favorable adsorption when 1< *n* < 10 [45]. PFO, proposed by Lagergren, assumes physical adsorption as the rate-controlling mechanism, while PSO assumes that the chemisorption controls the reaction rate [46,47,48].

The nonlinear regression was performed using OriginPro software, version 2020b, OriginLab Corporation, Northampton, MA, USA.

## 4. Conclusions

The simultaneous removal of heavy metals (Cu, Cd, Cr, Ni, Zn and Pb) from contaminated aqueous solutions using thermally treated zeolitic volcanic tuffs from Racoș (Romania) as adsorbent was studied. The selectivity of the natural zeolite (Ca-clinoptilolite type) for the sorption of the studied heavy metals cations followed, in general, the following trend: Pb > Cr > Cu > Zn > Cd > Ni. Heavy metals removal efficiencies were powerfully influenced by the particle size, the samples with smaller particle sizes (0–0.05 mm) being more efficient in metal removal. The amount of metal adsorbed per unit of zeolite mass increased with the metal concentration in the initial solution. Generally, no relevant changes were observed in the removal efficiency of the zeolitic volcanic tuffs thermally treated at 200 °C and 350 °C. Though, in the case of samples thermally treated at 550 °C, a small decrease in the removal efficiencies was observed. The Cd, Zn, Cu, Cr, Zn, and Ni ions sorption was best described by the Langmuir model according to the high values of the correlation coefficient. The results indicated that the pseudo-first-order kinetic model presented the best correlation to the experimental data. The total immobilization of heavy metals in zeolite is caused mainly by ion exchange, but also by chemisorption. The obtained results demonstrate that the zeolitic volcanic tuff from Racoș (Romania) is an efficient adsorbent for the removal of heavy metals from aqueous solutions, with removal efficiencies reaching 99%, in specific conditions of initial concentration in the solution, particle sizes and contact time.

## Figures and Tables

**Table 1:** Chemical composition (wt.%) of NZ1, NZ2 and thermally treated NZ samples.

Zeolite	SiO_2_	Al_2_O_3_	Na_2_O	K_2_O	CaO	MgO	Fe_2_O_3_	MnO	Others	Si/Al
NZ1	67.30	12.60	0.27	2.69	2.74	1.59	1.57	0.028	11.20	4.71
NZ1-200	67.93	12.42	0.27	2.74	2.56	1.48	1.59	0.028	10.98	4.82
NZ1-350	67.66	12.01	0.26	2.68	2.82	1.62	1.54	0.026	11.38	4.97
NZ1-550	68.71	10.42	0.27	2.48	2.58	1.49	1.29	0.023	12.74	5.81
NZ2	68.36	11.95	0.28	2.55	2.32	1.51	1.27	0.025	11.73	5.04
NZ2-200	67.10	11.88	0.29	2.41	2.45	1.48	1.36	0.026	13.00	4.98
NZ2-350	66.83	11.75	0.29	2.63	2.54	1.51	1.44	0.027	12.98	5.01
NZ2-550	66.52	10.74	0.27	2.74	2.49	1.88	1.51	0.023	13.83	5.46

**Table 2:** Concentrations of heavy metals in solid and aqueous extract of NZ1, NZ2 and thermally treated NZ samples (*n* = three parallel determinations).

Zeolite	Cu	Cd	Cr	Ni	Pb	Zn	Cu	Cd	Cr	Ni	Pb	Zn
	mg kg^−1^ (Solid Sample)						µg L^−1^ (Aqueous Extract)					
NZ1	2.03	<0.2	8.23	3.50	5.67	4.41	<4	<2	<7	<8	<10	<8
NZ1-200	1.86	<0.2	7.86	4.11	5.62	4.18	<4	<2	<7	<8	<10	<8
NZ1-350	1.83	<0.2	8.25	4.25	4.96	4.23	<4	<2	<7	<8	<10	<8
NZ1-550	1.93	<0.2	8.70	4.36	5.46	4.40	<4	<2	<7	<8	<10	<8
NZ2	1.88	<0.2	7.88	3.21	5.98	4.25	<4	<2	<7	<8	<10	<8
NZ2-200	1.77	<0.2	8.02	3.74	5.84	4.08	<4	<2	<7	<8	<10	<8
NZ2-350	1.65	<0.2	8.41	3.41	5.81	4.11	<4	<2	<7	<8	<10	<8
NZ2-550	1.71	<0.2	7.31	3.82	6.78	4.24	<4	<2	<7	<8	<10	<8

**Table 3:** Exchangeable cations content, the sum of exchangeable cations (CEC), surface area and pore volumes of the NZ1, NZ2 and thermally treated NZ samples (*n* = three parallel determinations).

Zeolite	Na^+^	K^+^	Ca^2+^	Mg^2+^	CEC	Surface Area	Average Pore Radius	Total Pore Volume
	mEq 100 g^−1^					m^2^ g^−1^	nm	cm^3^ g^−1^
NZ1	8.5	26.7	59.3	3.7	98.2	37	4–6; 25	0.05
NZ1-200	7.9	27.4	65.0	3.9	104.2	38	4–6; 25	0.06
NZ1-350	8.3	27.9	69.6	3.2	109.0	37	4–5; 25	0.06
NZ1-550	7.5	30.1	43.6	3.0	84.2	33	4–6; 36	0.06
NZ2	8.3	31.1	69.0	4.6	112.9	37	4–6; 25	0.05
NZ2-200	8.6	38.8	56.9	5.7	110.0	38	4–6; 25	0.06
NZ2-350	9.1	27.9	53.0	4.5	94.6	38	4–5; 25	0.06
NZ2-550	8.0	30.3	41.9	2.5	82.8	34	4–6; 33	0.06

**Table 4:** Langmuir and Freundlich isotherm parameters for Pb, Cd, Zn, Cu, Cr, and Ni ions sorption onto zeolite with a 0–0.05 mm particle size thermally treated at 200 °C.

Isotherm	Parameters	Pb	Cd	Zn	Cu	Cr	Ni
Langmuir	*q_max_* (mg g^−1^)	0.393	0.112	0.094	0.129	0.142	0.069
	*K_L_* (L mg^−1^)	12.318	44.204	114.045	73.447	64.873	47.306
	R^2^	0.9982	0.9615	0.9999	0.9814	0.9812	0.9930
Freundlich	*n*	0.526	0.098	0.071	0.082	0.092	0.057
	*K_F_* (mg^(1−1/*n*)^ L^1/*n*^ g^−1^)	0.615	0.092	0.081	0.102	0.108	0.060
	R^2^	0.9989	0.8414	0.7689	0.7077	0.7775	0.9312

**Table 5:** PFO and PSO kinetic parameters for Pb, Cd, Zn, Cu, Cr, and Ni ions sorption onto zeolite with a 0–0.05 mm particle size thermally treated at 200 °C.

Model	Concentration	Parameters	Pb	Cd	Zn	Cu	Cr	Ni
PFO	5 mg L^−1^	*q_e,calc_*	0.050	0.048	0.049	0.050	0.050	0.043
		*k*_1_	0.819	0.525	0.584	0.779	4867.509	0.323
		R^2^	0.9997	0.9965	0.9982	1.0000	0.9995	0.9500
	10 mg L^−1^	*q_e,calc_*	0.100	0.085	0.087	0.099	0.100	0.060
		*k*_1_	1.046	0.221	0.260	0.701	0.971	0.215
		R^2^	0.9999	0.9528	0.9881	0.9999	0.9999	0.9577
	30 mg L^−1^	*q_e,calc_*	0.298	0.109	0.092	0.125	0.128	0.064
		*k*_1_	0.408	0.261	0.716	0.481	0.500	0.191
		R^2^	0.9999	0.9728	0.9989	0.9994	0.9599	0.9717
PSO	5 mg L^−1^	*q_e,calc_*	0.050	0.049	0.049	0.050	0.050	0.046
		*k*_2_	149.370	37.503	51.840	174.468	741.378	12.578
		R^2^	0.8658	0.8173	0.8168	0.8497	0.0379	0.5796
	10 mg L^−1^	*q_e,calc_*	0.100	0.094	0.093	0.100	0.100	0.066
		*k*_2_	283.074	3.550	5.009	54.080	196.625	4.837
		R^2^	0.9996	0.7811	0.9179	0.9577	0.9980	0.8215
	30 mg L^−1^	*q_e,calc_*	0.306	0.117	0.093	0.128	0.129	0.070
		*k*_2_	4.207	3.879	45.229	13.338	7.789	3.880
		R^2^	0.8133	0.8752	0.7733	0.9877	0.3733	0.9152
Experimental data	5 mg L^−1^	*q_e,exp_*	0.050	0.050	0.050	0.050	0.050	0.049
	10 mg L^−1^	*q_e,exp_*	0.100	0.097	0.093	0.100	0.100	0.068
	30 mg L^−1^	*q_e,exp_*	0.297	0.118	0.093	0.126	0.113	0.070

**Table 6:** Desorption rate (%) of Pb, Cd, Zn, Cu, Cr, and Ni from zeolite with a 0–0.05 mm and 1.0–3.0 mm particle size thermally treated at 200 °C, after the sorption experiments from solutions of 5 mg L^−1^, 10 mg L^−1^, 30 mg L^−1^ and 60 min contact time.

Particle Size	Initial Concentration in Solution	Pb (%)	Cd (%)	Zn (%)	Cu (%)	Cr (%)	Ni (%)
1.0–3.0 mm	5 mg L^−1^	64.4	67.8	50.2	87.0	44.4	42.6
	10 mg L^−1^	63.5	66.4	51.3	85.6	41.9	36.8
	30 mg L^−1^	60.4	61.1	44.4	85.0	42.6	32.8
0–0.05 mm	5 mg L^−1^	70.5	70.5	59.8	88.7	46.7	43.2
	10 mg L^−1^	65.8	70.2	60.4	86.5	41.1	40.0
	30 mg L^−1^	63.0	66.2	55.8	85.8	42.9	38.4

**Table 7:** Nonlinear equations of Langmuir and Freundlich isotherms, PFO and PSO kinetic models.

Model	Equations	Description	Reference
Langmuir isotherm	$q_{e}=\frac{q_{max}K_{L}C_{e}}{1+K_{L}C_{e}}$	*q_max_* is the maximum adsorption capacity (mg g^−1^) and *K_L_* is the Langmuir constant (L mg^−1^)	[44,48]
Freundlich isotherm	$q_{e}=K_{F}C_{e}{}^{\left(1/n\right)}$	*K_F_* is related to adsorption capacity (mg^1−1/*n*^ L^1/*n*^ g^−1^) and 1/*n* is the adsorption intensity	[44,48]
PFO	$q_{t}=q_{e}\left(1-e^{-k_{1}t}\right)$	*q_t_* is the amount adsorbed at time *t* (mg g^−1^) and *k*_1_ is the first-order rate constant (min^−1^)	[44,48]
PSO	$q_{t}=\frac{q_{e}^{2}k_{2}t}{1+q_{e}k_{2}t}$	*k*_2_ is the second-order rate constant (g mg·min^−1^)	[44,48]

## Data Availability

Not applicable.

## Sample Availability

Samples are available from the authors.
